# Predicting IDH and ATRX mutations in gliomas from radiomic features with machine learning: a systematic review and meta-analysis

**DOI:** 10.3389/fradi.2024.1493824

**Published:** 2024-10-31

**Authors:** Chor Yiu Chloe Chung, Laura Elin Pigott

**Affiliations:** ^1^ Institute of Health and Social Care, London South Bank University, London, United Kingdom; ^2^ Department of Brain Repair and Rehabilitation, Queen Square Institute of Neurology, University College London, London, United Kingdom

**Keywords:** machine learning, glioma, radiomics, MRI, ATRX, IDH, neuroimaging

## Abstract

**Objective:**

This systematic review aims to evaluate the quality and accuracy of ML algorithms in predicting ATRX and IDH mutation status in patients with glioma through the analysis of radiomic features extracted from medical imaging. The potential clinical impacts and areas for further improvement in non-invasive glioma diagnosis, classification and prognosis are also identified and discussed.

**Methods:**

The review followed the Preferred Reporting Items for Systematic Reviews and Meta-Analyses of Diagnostic and Test Accuracy (PRISMA-DTA) statement. Databases including PubMed, Science Direct, CINAHL, Academic Search Complete, Medline, and Google Scholar were searched from inception to April 2024. The Quality Assessment of Diagnostic Accuracy Studies (QUADAS-2) tool was used to assess the risk of bias and applicability concerns. Additionally, meta-regression identified covariates contributing to heterogeneity before a subgroup meta-analysis was conducted. Pooled sensitivities, specificities and area under the curve (AUC) values were calculated for the prediction of ATRX and IDH mutations.

**Results:**

Eleven studies involving 1,685 patients with grade I–IV glioma were included. Primary contributors to heterogeneity included the MRI modalities utilised (conventional only vs. combined) and the types of ML models employed. The meta-analysis revealed pooled sensitivities of 0.682 for prediction of ATRX loss and 0.831 for IDH mutations, specificities of 0.874 and 0.828, and AUC values of 0.842 and 0.948, respectively. Interestingly, incorporating semantics and clinical data, including patient demographics, improved the diagnostic performance of ML models.

**Conclusions:**

The high AUC in the prediction of both mutations demonstrates an overall robust diagnostic performance of ML, indicating the potential for accurate, non-invasive diagnosis and precise prognosis. Future research should focus on integrating diverse data types, including advanced imaging, semantics and clinical data while also aiming to standardise the collection and integration of multimodal data. This approach will enhance clinical applicability and consistency.

## 1 Introduction

Gliomas are the most common type of primary malignant tumours, accounting for approximately 77% of cases, with 23% including other types such as meningiomas, medulloblastomas and pituitary adenomas. Gliomas are classified into grades I–IV based on histopathological and molecular analysis according to the World Health Organization (WHO) (1). Survival rates for patients with low-grade glioma vary significantly, from 2 to 12 years, depending on the age of diagnosis, tumour location and histologic type (2). In contrast, patients with aggressive grade IV gliomas typically survive less than 2 years despite treatment advancements (3). An abbreviation and terminology explanations table can be found in Supplementary Figure S1.

Isocitrate dehydrogenase (IDH) and α-thalassemia/mental retardation syndrome X-linked gene (ATRX) are key biomarkers used for the analysis and classification of gliomas. Grading of gliomas has traditionally been solely based on histological features. However, grading now incorporates biomarker statuses such as IDH and ATRX per the 2016 WHO classification, which was revised in 2021 (1). Beyond the grading of gliomas, IDH and ATRX mutation statuses provide valuable insight and crucial prognostic information to support decision-making and treatment planning. IDH mutations are often associated with a better prognosis compared to IDH wildtype. ATRX loss generally promotes cancer cell survival by activating the alternating lengthening telomere (ALT) pathway; however, recent research suggests that when combined with IDH mutations, ATRX loss paradoxically correlates with improved prognosis. This is attributed to enhanced immune responses and increased genomic instability, which collectively contribute to better survival rates (4–7).

Neuroimaging techniques, including magnetic resonance imaging (MRI), positron emission tomography (PET) and computed tomography (CT) scans, are essential for the non-invasive identification and monitoring of glioma (8). Recent advancements in neuroimaging, including dynamic susceptibility contrast, diffusion- and perfusion-weighted imaging (DSC, DWI and PWI) have significantly improved glioma characterisation and molecular profiling (9, 10). Radiomics is the analysis of medical imaging and often employs machine learning (ML) to extract quantifiable image-based features, indicating structural alterations and pathophysiological processes (11–13). Non-invasive imaging can assess the entire tumour, providing advantages over biopsy and resection. Although histopathological testing remains the gold standard for definitive diagnosis, the invasiveness of the procedure leads to inherent risks including infection, bleeding and possible restriction by sampling error. Additionally, incorporating ML can identify patterns in medical imaging potentially missed by clinical interpretation alone, thereby improving diagnostic accuracy and patient outcomes **(**14). These advancements offer the potential for accurate, efficient and non-invasive diagnostic approaches. However, challenges include variations in imaging modalities and ML approaches, which may influence the accuracy and outcomes (15, 16).

Previous reviews have focused on the accuracy of ML in predicting biomarkers including IDH, MGMT and 1p19q (11, 17, 18). The most recent studies included in a systematic review on predicting IDH and MGMT statuses using ML and radiomic features were published in September 2021 (19). Another study reviewed the accuracy of radiomics in predicting IDH mutations, specifically in diffuse gliomas (20). Nonetheless, newer research has emerged, and a systematic review has yet to comprehensively evaluate the potential of using ML to predict both ATRX and IDH mutation statuses from extracted radiomic features, which together may be associated with more positive patient prognosis.

Given the mutual prognostic implications of these mutations, this review aims to identify and synthesise the most current studies to evaluate the diagnostic accuracy of ML in predicting ATRX and IDH mutation statuses. The review will also assess the potential and clinical impact of using ML algorithms (MLA) for non-invasive and efficient glioma treatment planning, prognosis and overall patient care.

## 2 Materials and methods

### 2.1 Search strategy and study selection

The Preferred Reporting Items for Systematic Reviews and Meta-Analyses of Diagnostic and Test Accuracy (PRISMA-DTA) statement was adhered to for this systematic review and meta-analysis (21). The research question was formulated using the Population, Exposure and Outcome (PEO) framework (Table 1). A comprehensive search was conducted across major databases including PubMed, CINAHL, Academic Search Complete, Medline, Science Direct, and Google Scholar for grey literature. The search strategy included “machine learning”, “glioma”, “ATRX mutation”, “IDH mutation” and related terms to identify research from database inception to April 2024 (Table 2). The search strategy and screening process for study abstracts and full texts were independently carried out by two reviewers, to ensure the comprehensiveness and accuracy of the selection process. Any disagreements were resolved through discussion.

**Table 1:** PEO research framework.

Population	Exposure	Outcome
Patients with a grade II–IV glioma diagnosis	Analyses of radiomic features using ML algorithms to predict IDH and ATRX mutation status	Effectiveness and accuracy of ML algorithms in determining IDH and ATRX mutation status from radiomic features

**Table 2:** Table portraying the search terms, combinations and Boolean operators included in the search strategy.

Search No.	Search term
1	“Machine Learning” AND “Glioma” AND “ATRX” AND “IDH”
2	“Machine learning” OR “Artificial Intelligence” OR “Deep Learning” OR “Neural Network”
3	“IDH” OR “Isocitrate Dehydrogenase” OR “IDH1”
4	“Glioma” OR “Brain Tumour” OR “Glioblastoma”
5	"ATRX” OR “alpha-thalassemia/mental retardation, X-linked"
6	(#2) AND (#3) AND (#4) AND (#5)

Eligibility criteria were predetermined using the Population, Intervention, Comparison, Outcome and Time (PICOT) framework (Table 3). The included studies were required to use neuroimaging as part of the ML approach in predicting IDH and ATRX mutation.

**Table 3:** The eligibility table displays the predefined inclusion and exclusion criteria and justification following the PICOT framework.

	Inclusion criteria	Exclusion criteria	Justification
Population (P)	Adult patients diagnosed with grade I–IV glioma	Non-human research	To ensure relevance to the research question and objective
Intervention (I)	MLA is used to predict both ATRX and IDH status using neuroimaging data	ML was not used to predict both ATRX and IDH mutation status	To ensure focus on evaluating the effectiveness of MLA with neuroimaging
Comparison (C)	IDH mutations and ATRX loss confirmed via histopathological and/or molecular testing	Studies that did not use histopathological and/or molecular testing.	Gold standards in confirming mutation status for patients with glioma for a standard reference
Outcome (O)	Accuracy metrics for ML reported including sensitivity, specificity and AUC	Studies that did not report these accuracy metrics	To allow evaluations of the diagnostic performance of ML algorithms
Timeframe (T)	Conducting searches from inception to present	N/A	To capture all relevant research developments over the recent years regarding the clinical use of ML
Study design	Primary research.	Reviews, editorials, letters and conference abstracts only	Ensuring comprehensive and original research data is included only
		Non-English studies and duplications	Duplicate will not contribute any new information

AUC, area under the curve; MLA, machine learning algorithms.

### 2.2 Data extraction and quality appraisal

A data extraction form was used to extract relevant information, which was predetermined before extraction to minimise bias, from the included studies (Supplementary Figure S2).

The Quality Assessment of Diagnostic Accuracy Studies (QUADAS-2) tool was used to assess the risk of bias (RoB) and concerns regarding applicability. This consisted of a comprehensive assessment across four domains including patient selection, index tests, reference standards and the flow and timing of the study with a completed example shown in Supplementary Figure S3 (22). To further enhance the appraisal of study quality and improve the significance of the results, the METhodological RadiomICs Score (METRICS), which is a novel tool developed specifically for radiomics research, was employed. The METRICS tool enabled a comprehensive evaluation of the methodologic quality of the assessed research papers across 30 items and nine domains including study design, imaging data, image processing and feature extraction, metrics and comparison, testing, feature processing, preparation for modelling, segmentation and open science (23).

### 2.3 Statistical analysis

The primary outcome of this systematic review is to evaluate the accuracy of using ML to predict ATRX and IDH mutation status from extracted radiomic features. This includes analysing accuracy metrics such as pooled sensitivity, specificity and area under the curve (AUC)**.**

#### 2.3.1 Meta-analysis

As the raw data were not reported in all studies, confusion matrices (2 × 2 tables) were reconstructed (Supplementary Table S1 and S2), to calculate pooled sensitivity and specificity (24). For studies reporting multiple results from training and test (validation) sets, data from the test sets were used for analysis. Meta-analysis was conducted using OpenMeta[Analyst] software (MetaAnalyst, Brown University EPBC), which uses R packages for statistical analysis (25).

The Chi-square and Higgins inconsistency index (*I*^2^) tests were conducted to assess for heterogeneity. In the Chi-square test, *p* < 0.05 indicates the presence of heterogeneity. The *I*^2^ statistic was used to evaluate the degree of heterogeneity, following the interpretation guidelines from the Cochrane Handbook for Systematic Reviews of Interventions: *I*^2^ = 0%–40%, heterogeneity might not be important; 30%–60%, heterogeneity may be moderate; 50%–90%, heterogeneity may be substantial; and 75%–100%, considerable heterogeneity. Pooled estimates for sensitivity, specificity and AUC and 95% confidence intervals (CI) were calculated using a random-effects model due to expected heterogeneity among studies regarding methodology between studies (26).

#### 2.3.2 Meta-regression and subgroup analysis

Meta-regression was conducted using IBM SPSS Statistics (IBM Corp. Released 2017. IBM SPSS Statistics for Windows, Version 25.0. Armonk, NY, USA: IBM Corp.) to determine covariates contributing to heterogeneity. The covariates included the number of extracted radiomic features; the mean age of the patient group; the number of mutations; the sample size; the types of ML models, which were support vector machine (SVM) or tree-based or convolutional neural network (CNN)-based or others; and the MRI modality, which was conventional only or combined. A subgroup analysis was subsequently conducted based on findings from the meta-regression to reduce heterogeneity (26).

Less than 10 studies were included in the meta-analysis. Therefore, publication bias was not assessed, due to the low power of the tests, which may lead to inconclusive results from the funnel plots for detecting publication bias (27).

## 3 Results

### 3.1 Study selection

**Figure 1:**
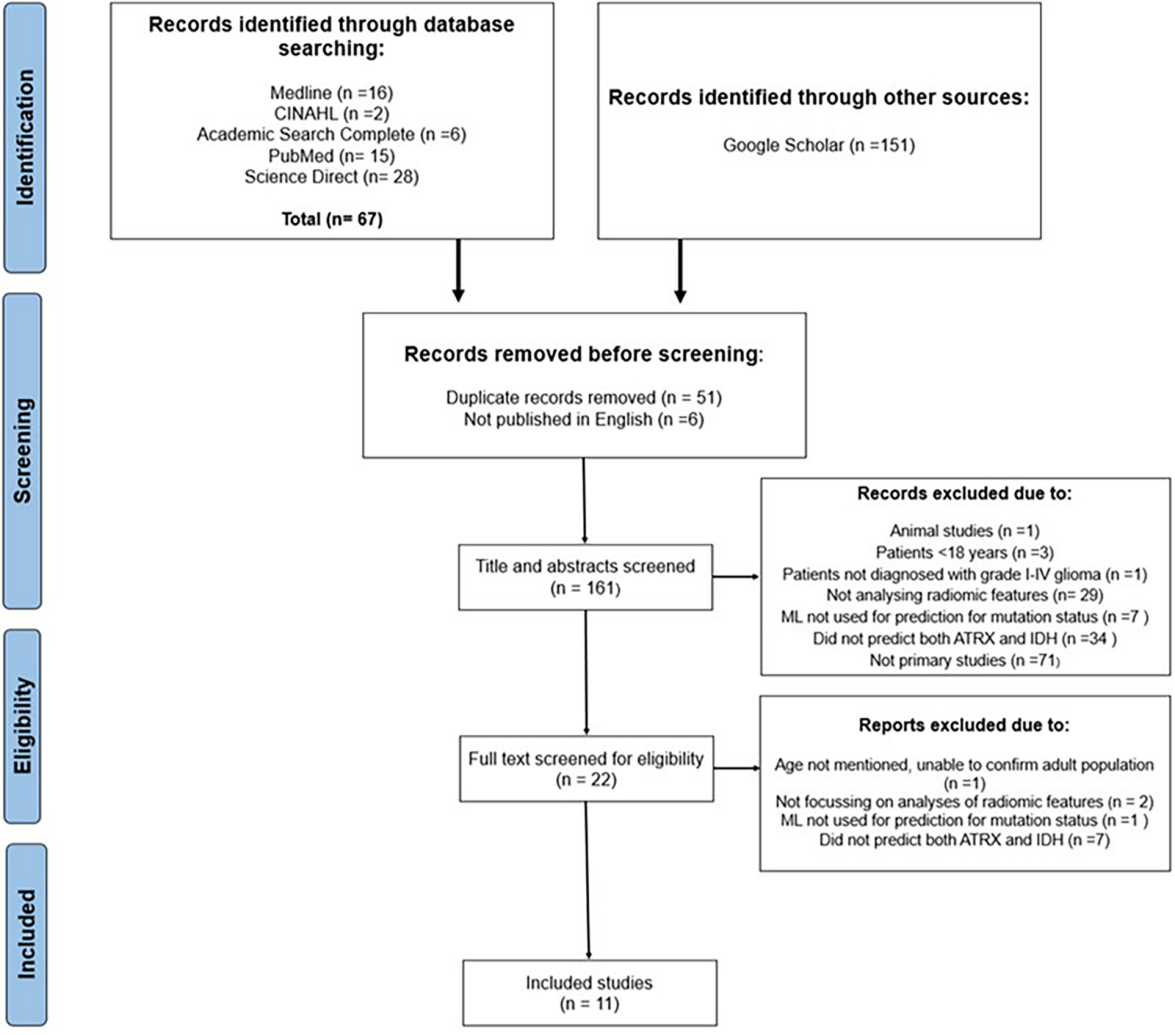
PRISMA flowchart illustrating the sifting and selecting process for the systematic review.

A total of 218 publications were initially retrieved with 161 remaining after duplicates and non-English publications were removed. Moreover, 139 results were excluded after titles and abstracts were screened, which resulted in 22 full texts being assessed for eligibility. Finally, 11 studies were included in the systematic review (Figure 1).

### 3.2 Study characteristics and outcomes

This systematic review included 1,685 patients with grade I–IV glioma across the 11 studies. Studies either utilised conventional MRI only or a multiparametric approach combining both conventional and advanced MRI techniques (Table 4).

**Table 4:** Study and patient characteristics tables. Including patient demographics and study characteristics detailing MLA and radiomics used in the included studies.

Study ID	Study design	Glioma grade	Mean age	Sample size	No. of features extracted	MLA	Radiomic features analysed
Ren et al. (28)	RO	II	41.1	57	260	SVM	T2FLAIR, CBF, ADC, eADC
Haubold et al. (29)	PO	I–IV	49.3	30	19,284	SVM and RF	Multiparametric
Calabrese et al. (30)	RO	IV	Adults, no mean age given	199	5,300	RF	T1, 3D ASL, 3D T2, SWI, DWI, T2W FLAIR, 2D HARDI
Shboul et al. (14)	RO	II–III	46.5	108	680	XGBoost	T1, T2, T2 FLAIR
Haubold et al. (31)	RO	II–IV	50.2	217	4,686	XGBoost, Boruta	T1, FLAIR
Sohn et al. (32)	RO	IV	60.1	126	660	BR, EC and SVM	T1, T2, FLAIR
Calabrese et al. (33)	RO	IV	60.0	400	5,300	CNN and RF	T1, T2, T2 FLAIR, SWI, ASL, DWIs, HARDI
Wu et al. (34)	RO	II–III	43.5	111	250	Elastic Net Regression	Multiparametric
Musigmann et al. (35)	RO	I–IV	43.5	124	107	AutoML	T2
Rui et al. (36)	RO	II–IV	47.0	42	NA	CNN	QSM, 3.0 T MRI, T2FLAIR, T1 + C
Zhong et al. (37)	RO	IV	48.0	37	428	ResNet50 and C3D	T1, T1 + C, T2

MLA, machine learning algorithms; PO, prospective observational; RO, retrospective observational. Full abbreviations and explanations table can be found in Supplementary Figure S1.

Sensitivity, specificity and AUC for ML in predicting IDH mutation ranged from 0.69 to 1.00, 0.67 to 0.94 and 0.74 to 0.98, respectively. The highest sensitivity, specificity and AUC achieved were 1.00 (28, 32), 0.94 (33) and 0.98 (37), respectively.

Sensitivity, specificity and AUC for the prediction of ATRX loss ranged from 0.53 to 0.97, 0.53 to 0.95 and 0.60 to 0.97, respectively. The highest sensitivity, specificity and AUC were 0.97 (33), 0.95 (32) and 0.97 (30), respectively (Table 5).

**Table 5:** Summary of ML performance in prediction of IDH and ATRX mutations from included studies.

Study ID	IDH sensitivity	IDH specificity	IDH AUC	ATRX sensitivity	ATRX specificity	ATRX AUC
Ren et al. (28)	1.00	0.86	0.93	0.95	0.88	0.93
Haubold et al. (29)	0.77	0.87	0.89	0.84	0.75	0.85
Calabrese et al. (30)	0.93	0.88	0.95	0.94	0.92	0.97
Shboul et al. (38)	0.90	0.79	0.84	0.69	0.83	0.70
Haubold et al. (31)	0.69	0.80	0.86	0.66	0.85	0.92
Sohn et al. (32)	1.00 (BR) 1.00 (ECC)	0.88 (BR) 0.88 (ECC)	0.96 (BR) 0.97 (ECC)	0.53 (BR) 0.70 (ECC)	0.95 (BR) 0.85 (ECC)	0.78 (BR) 0.82 (ECC)
Calabrese et al. (33)	0.86	0.94	0.96	0.97	0.88	0.97
Wu et al. (34)	N/A	N/A	0.90	N/A	N/A	0.84
Musigmann et al. (35)	N/A	N/A	0.74	N/A	N/A	0.76
Rui et al. (36)	0.86	0.67	0.77	0.65	0.53	0.60
Zhong et al. (37)	0.77 (3DResNet) 0.90 (C3D)	0.74 (3DResNet) 0.84 (C3D)	0.98	0.79 (3DResNet) 0.89 (C3D)	0.84 (3DResNet) 0.91 (C3D)	0.95

### 3.3 Quality appraisal and risk of bias

**Figure 2:**
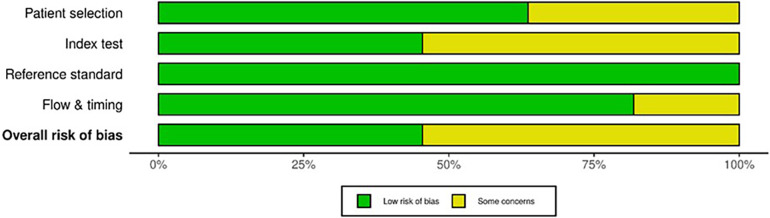
Summary of RoB and quality assessed using QUADAS-2.

**Figure 3:**
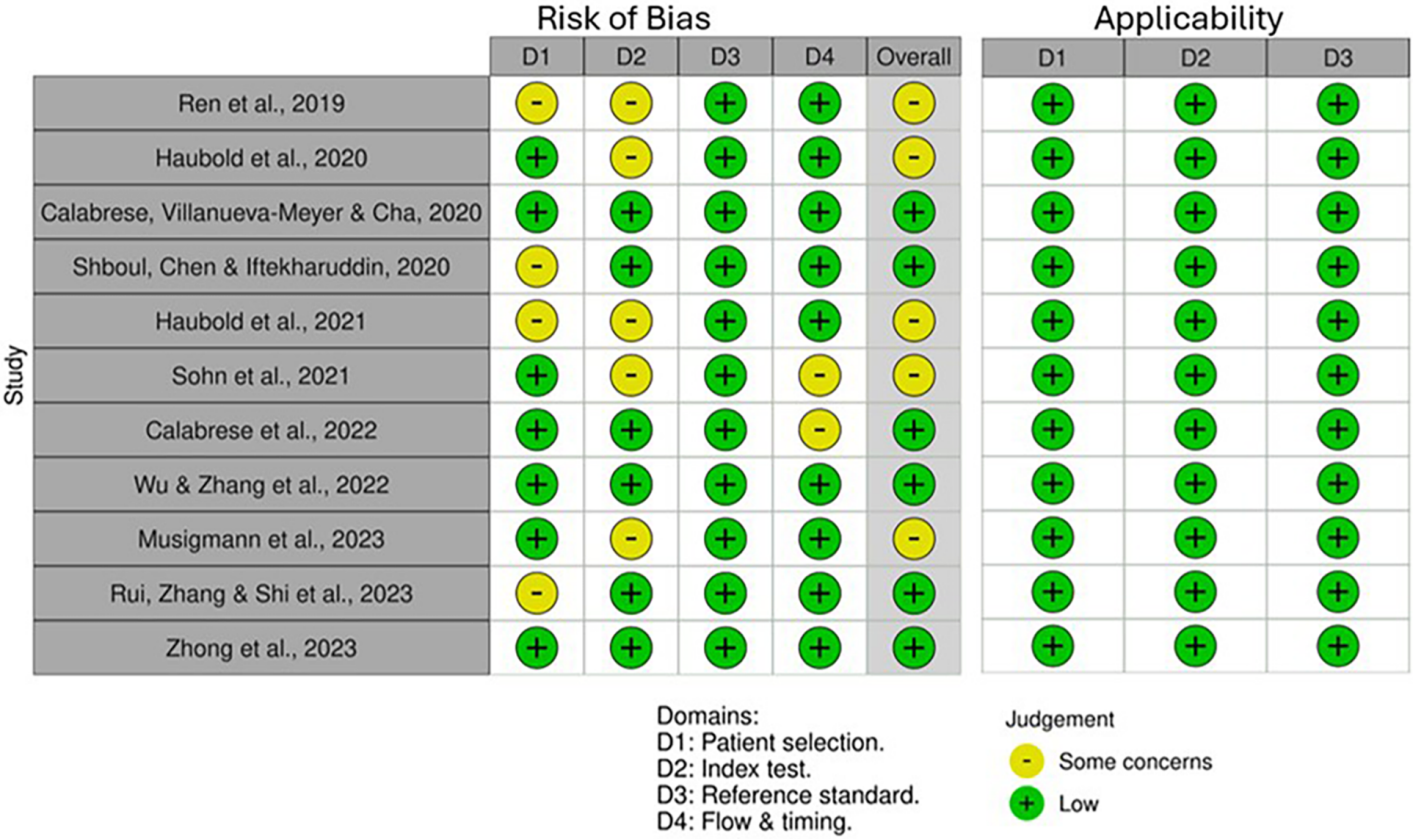
Traffic light plot detailing the assessed domains in QUADAS-2 for each included study.

QUADAS-2 revealed varying RoB across the four domains (Figure 2). Some studies did not mention whether a random or consecutive selection process was used, leading to unclear RoB for Domain 1. Concerns were also noted in Domain 2 due to unclear reporting on threshold pre-specification and blinding during test interpretation. Furthermore, all studies used histopathological examination as the reference standard, resulting in consistently low RoB for Domain 3. External validation in studies by Calabrese et al. (30) and Zhong et al. (37) improved quality and reduced bias. Applicability for all included studies was low concerning patient selection, index test and standard reference (Figure 3).

**Figure 4:**
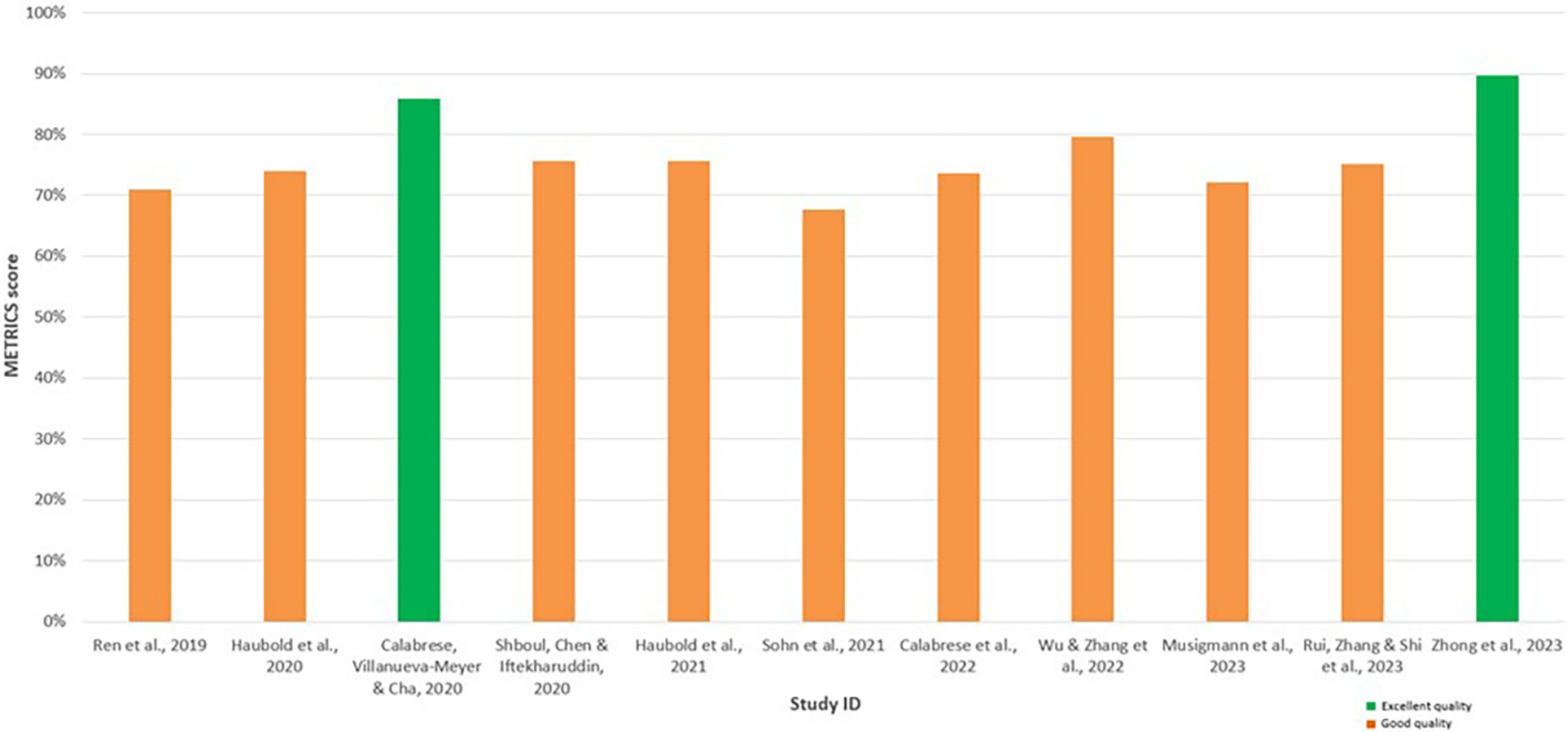
Bar chart displaying the METhodological RadiomICs (METRICS) scores for each assessed radiomic study, facilitating comparison. The green bars indicate the studies appraised as having excellent quality, while the orange bars indicate the studies with good quality.

METRICS revealed similar results, thereby enhancing the significance of the findings, with all studies appraised as having good or excellent quality (Table 6). Notably, Calabrese et al. (30) and Zhong et al. (37) were also evaluated as the highest quality among the included radiomics studies using the METRICS tool, achieving an excellent quality category for both studies with METRICS scores of 85.9% and 89.7%, respectively (Figure 4).

**Table 6:** Results for METRICS appraisal of included studies.

Study	METRICS score	Quality category
Ren et al. (28)	71.0%	Good
Haubold et al. (29)	74.0%	Good
Calabrese et al. (30)	85.9%	Excellent
Shboul et al. (14)	75.6%	Good
Haubold et al. (31)	75.6%	Good
Sohn et al. (32)	67.7%	Good
Calabrese et al. (33)	73.7%	Good
Wu et al. (34)	79.6%	Good
Musigmann et al. (35)	72.2%	Good
Rui et al. (36)	75.1%	Good
Zhong et al. (37)	89.7%	Excellent

This table evaluates 30 items across nine domains to assess the methodological rigour of the radiomic studies, resulting in a METRICS score and quality category for each study.

### 3.4 Heterogeneity assessment and meta-regression

The initial meta-analysis including all 11 studies showed homogeneity in the sensitivity outcomes between studies (*p* = 0.156, *I^2^* = 31.59%). Nonetheless, considerable heterogeneity was revealed in specificity (*p* < 0.001, *I^2^* = 77.96%) and AUC (*p* < 0.001, *I^2^* = 98.29%) for predicting IDH mutations. For predicting ATRX loss, moderate heterogeneity was observed for sensitivity (*p* = 0.038, *I^2^* = 49.44%) and specificity (*p* = 0.009, *I^2^* = 59.19%), and considerable heterogeneity was found for AUC (*p* < 0.001, *I^2^* = 99.29%) (26).

**Figure 5:**
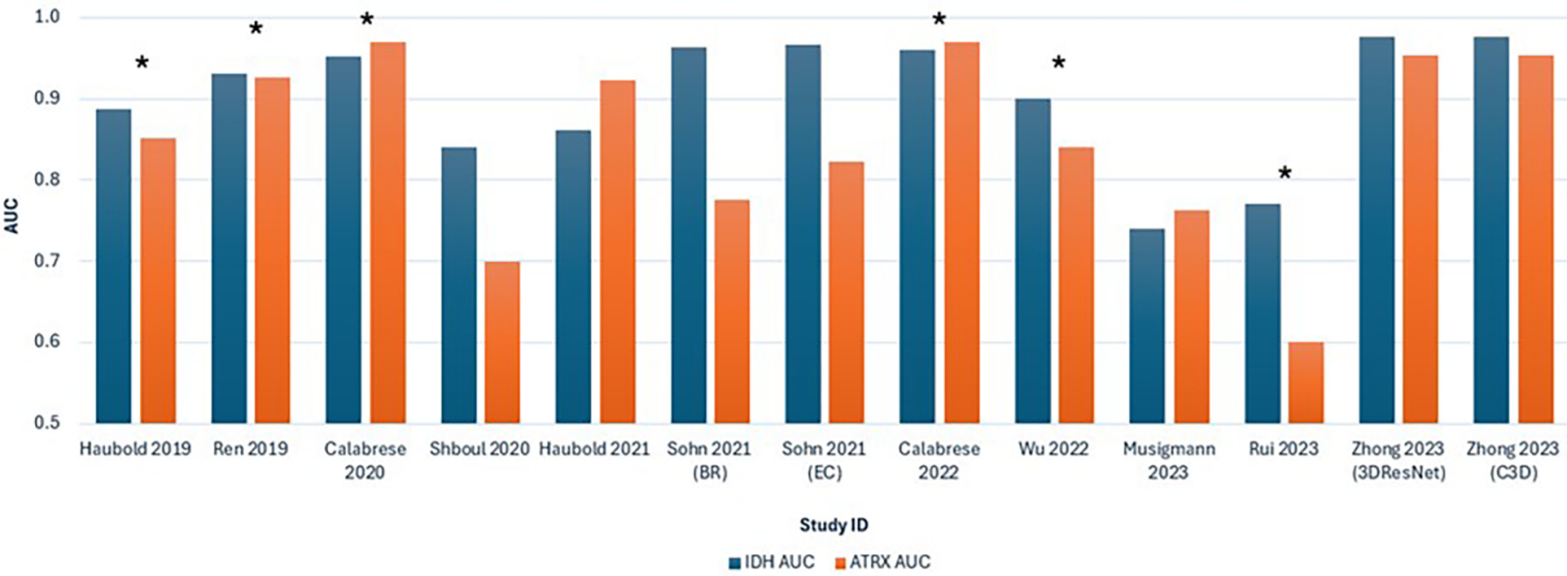
Bar chart comparing the AUC values for predicting IDH and ATRX mutations across the 11 included studies. Each study is represented by two bars, with blue indicating IDH AUC and orange indicating ATRX AUC. *Studies that combined advanced and conventional MRI modalities.

Figure 5 shows a graphical interpretation demonstrating the variability in the AUC for ML performance across the studies, especially for predicting ATRX loss, which aligns with the assessed heterogeneity. Additionally, significant variability is shown between studies that use multiparametric approaches. The ML model in Rui et al. (36) performs visibly poorer in predicting IDH and ATRX mutation compared to other studies.

The results from the meta-regression show that heterogeneity was influenced by the number of extracted radiomic features (*p* = 0.009), type of ML models (*p* < 0.001) and MRI modality used (*p* < 0.001). Other assessed covariates including the mean age of patients (*p* = 0.073) and the number of patients included (*p* = 0.073) did not significantly contribute to the observed heterogeneity.

### 3.5 Subgroup meta-analysis

Subgroup analyses were performed based on the results of the meta-regression. Therefore, for analysis of sensitivity and specificity, studies using a combination of conventional and advanced MRI were excluded. Furthermore, the meta-analysis of AUC only included studies that provided sufficient data by reporting CI for consistent and accurate calculation of standard error (SE).

#### 3.5.1 Predicting IDH mutation status

**Figure 6:**
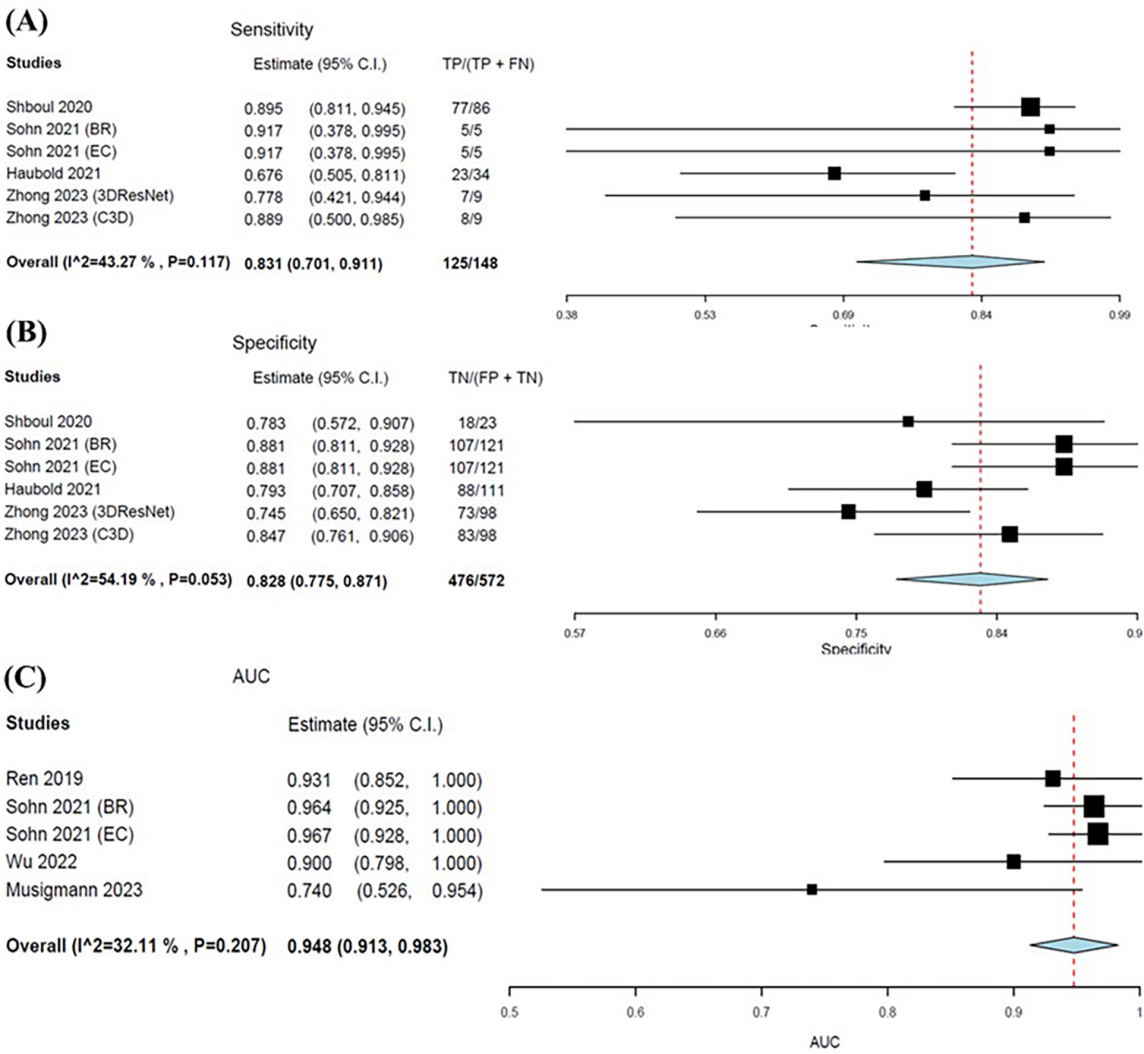
Forest plots showing the sensitivity **(A)**, specificity **(B)**, and AUC **(C)** of MLAs in predicting IDH status. Overall pooled data are presented at the bottom left of the forest plots with the values for Higgins *I*^2^ and Chi-square shown in brackets.

Four studies were eligible for inclusion with six outcomes synthesised and analysed in the meta-analyses. Sohn et al. (32) and Zhong et al. (37) evaluated two different models, and therefore each consisted of two outcomes for sensitivity, specificity and AUC. The pooled sensitivity and specificity were 0. 831 (95% CI: 0.701–0.911) and 0.828 (95% CI: 0.761–0.871), respectively (Figures 6A,B). Meta-analysis of AUC consisted of four studies with five outcomes. The pooled AUC for ML models in predicting IDH mutations was 0.948 (95% CI: 0.913–0.983) (Figure 6C). Additionally, the forest plots for all metrics graphically portrayed moderate heterogeneity, therefore further justifying the random-effects model used for the meta-analysis.

#### 3.5.2 Predicting ATRX mutation status

**Figure 7:**
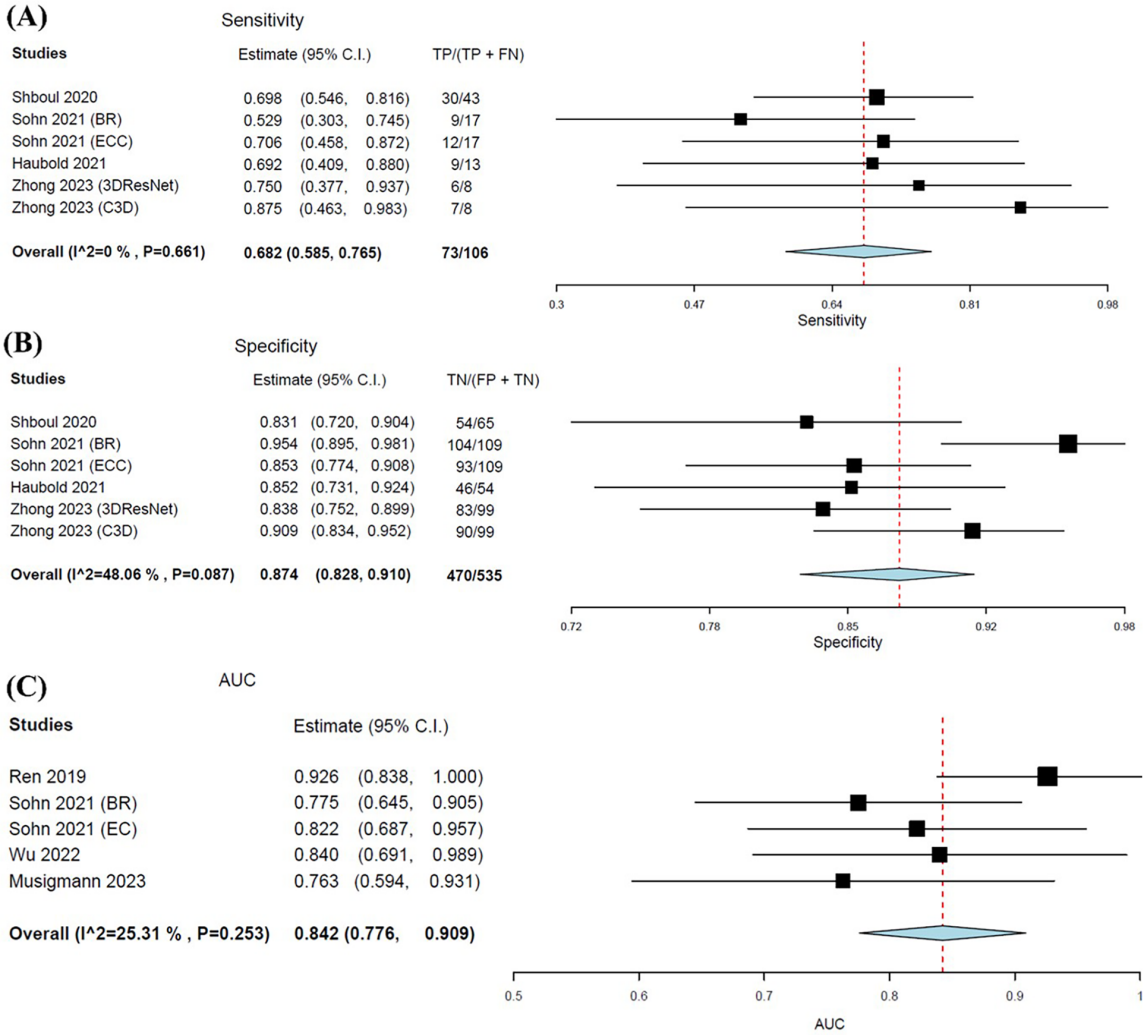
Forest plots showing the pooled sensitivity **(A)**, specificity **(B)**, and AUC **(C)** of MLA in predicting ATRX status. Overall pooled data are presented at the bottom left of the forest plots, with the values for Higgins *I*^2^ and Chi-square shown in brackets.

The pooled sensitivity was notably lower in predicting ATRX loss compared to IDH, at 0.682 (95% CI: 0.585–0.765). Whereas pooled specificity was slightly higher at 0.874 (95% CI: 0.828–0.910) (Figures 7A,B). The pooled AUC value for MLAs in predicting ATRX loss was 0.842 (95% CI: 0.776–0.909) (Figure 7C). The forest plots for all metrics graphically portrayed the presence of heterogeneity, particularly for specificity and AUC.

### 3.6 Incorporating clinical information and semantics

Wu et al. (34) developed a predictive nomogram model incorporating age, gender and radiomics signature. The odds ratio (OR) and 95% CI from univariate regression analysis determined the correlation of these predictors with IDH and ATRX mutations. Age was found to be a significant predictor for IDH mutations, with younger age associated with a higher probability (OR = 0.935, 95% CI: 0.894–0.978, *p* = 0.003). A high radiomics signature was a strong predictor for both IDH mutation (OR = 16.463, 95% CI: 4.898–55.338, *p* < 0.0001) and ATRX loss (OR = 24.676, 95% CI: 5.073–120.029, *p* < 0.0001). Although gender was not significant according to univariate logistic regression, gender was included in the multivariable model for its clinical importance. The decision curve analysis (DCA) demonstrated the clinical utility of the nomograms, which included all three variables. This led to the development of highly accurate nomograms with C-index values of 0.90 and 0.84 for predicting IDH and ATRX mutations, respectively, in the validation cohort. These findings highlight the importance of demographic information in predicting biomolecular status in gliomas and improving model performance.

Zhong et al. (37) incorporated semantic features such as tumour shape, location and heterogeneity into convolutional neural network (CNN)-based models. This resulted in substantial improvements in accuracy in predicting IDH and ATRX mutations, with increases from 85.56% to 91.11% and from 82.29% to 86.46%, respectively. Similarly, Calabrese et al. (30) used a deep learning-based automated segmentation algorithm to distinguish various elements of glioblastoma, such as enhancing and non-enhancing tumours, as well as surrounding oedema. Subsequently, this supports the importance of including qualitative imaging for accurately identifying IDH mutations.

### 3.7 External validation of predictive models

Zhong et al. (37) used external validation to confirm the robustness of the deep learning models, therefore ensuring the consistent reliability of ML models in predicting IDH and ATRX mutations across different datasets. High and moderate to low accuracy were achieved in the external validation for predicting IDH and ATRX mutation status, respectively. Accuracies of 83.51% and 88.30% were achieved for IDH mutations, whereas accuracies of 66.67% and 76.67% were achieved for ATRX loss with the 3DResNet and C3D integrated models, respectively.

Similarly, Calabrese et al. (30) also conducted an external validation and achieved relatively poor performance on the external compared to the internal dataset with an AUC of 0.63 for IDH and 0.72 for ATRX contrasting with 0.95 for IDH and 0.97 for ATRX, respectively.

## 4 Discussion

### 4.1 Main findings

This systematic review evaluated the effectiveness of ML in predicting IDH and ATRX mutations in gliomas using extracted radiomic features. Meta-analysis revealed pooled sensitivities of 0.682 for ATRX loss and 0.831 for IDH mutations, indicating higher accuracy for MLAs in identifying IDH mutations. ML models demonstrated high pooled specificities of 0.874 for ATRX loss and 0.828 for IDH mutations, with AUC values of 0.842 and 0.948, respectively. The high specificity indicates the strong capabilities of ML to accurately identify glioma patients without mutations, minimising false positives and aiding appropriate decisions on personalised treatment plans. The high AUC highlight the overall great diagnostic performance of ML models.

The review highlights the excellent diagnostic accuracy of ML models for IDH while emphasising the need to improve ATRX detection. This aligns with results from existing literature, where research reporting a high diagnostic performance for predicting IDH mutations has increased significantly since 2017 (11, 17, 39). Jian et al. (18) reported high diagnostic performance for IDH mutations with pooled sensitivity, specificity and AUC of 0.85, 0.83 and 0.90, respectively. However, for ATRX, more varied sensitivity and specificity were reported, ranging from 0.84 to 0.95 and 0.75 to 0.90, respectively. Lost et al. (19) also found a high mean AUC of 0.89 for IDH mutation prediction and a lower mean AUC of 0.72 for ATRX loss prediction.

### 4.2 Heterogeneity

Significant heterogeneity among the included studies was present. The meta-regression identified that covariates significantly contributing to heterogeneity included the different MRI modality combinations and the ML model types (*p* < 0.001). Figure 5 shows that ML models in three out of six studies using combined MRI approaches achieved AUC above 90% in predicting both mutations. Enhanced diagnostic accuracy by combining advanced with conventional MRI modalities was supported by recent literature. Incorporating advanced techniques can more comprehensively capture tumour characteristics and ultimately contribute to more accurate predictions (40, 41). Despite these advances in research regarding multiparametric approaches, conventional MRI remains prevalent in clinical practice due to its availability and standardised application (42, 43). Nonetheless, the predictive performance of ML models using multiparametric MRI varies and may be lower or similar to studies using conventional MRI, as shown in this review, which aligns with other recent reviews (44).

The diversity in ML models developed, from SVM and RF to CNN, also significantly contributes to heterogeneity. Different MLAs differ in feature extraction and model training approaches, which influence the performance of the ML models. No single MLA has shown to be superior, as suggested by the variability in predictive capability across all 11 studies (Figure 5).

### 4.3 Overall effectiveness of ML in predicting ATRX and IDH

The high diagnostic accuracy, particularly for identifying IDH mutations, demonstrated the excellent potential for effective use of ML models in non-invasive diagnosis for gliomas. The high AUC value of 0.948 for predicting IDH mutations emphasises the robust capability of ML models to provide reliable predictions for ATRX loss and IDH mutations. This aligns with studies by Jian et al. (18) and Karabacak et al. (45), who have reported similarly strong performance in predicting IDH mutations with AUC values of 0.90 and 0.89, respectively. These results demonstrate great potential in the integration of ML models into clinical practice to offer reliable prediction for IDH mutation status.

However, the overall performance for the prediction of ATRX mutations shows greater variability compared to the prediction of IDH mutations across studies. Compared to the pooled AUC of 0.842 for predicting ATRX mutation status identified in this review, Lost et al. (19) reported a lower mean AUC of 0.72, while Mora et al. (46) also achieved a high performance with an AUC of 0.831. These findings suggest promise in incorporating ML with radiomics to predict ATRX predictions; nonetheless, further research and optimisation will be essential to enhance and ensure consistent performance.

### 4.4 The impact of incorporating clinical information and semantics on ML performance

The review revealed that incorporating clinical information and semantic features improves the accuracy of ML in predicting IDH and ATRX mutations. The findings were consistent with recent reviews that demonstrated enhanced performance via multimodal data fusion with the incorporation of clinical characteristics, demographics and semantic features (47).

Primary studies also supported the inclusion of clinical and semantic data in ML models for glioma diagnosis and prognosis (39, 48). Kazerooni et al. (39) emphasized the potential of integrated diagnostics by exploring the use of multi-omics, which combines radiomics, molecular status and clinical measures. This approach yielded superior performance in predicting overall survival in glioblastoma patients, resulting in more comprehensive patient profiles to facilitate personalised treatment planning. Similarly, Jang et al. 2020 also integrated radiomic features with clinical information to distinguish pseudoprogression from true glioma progression. This provides valuable insights into the broader application of multimodal data and highlights the potential to reduce the need for multiple imaging modalities, which may lead to substantial memory usage and the “curse of dimensionality”. This term refers to the issue of having too many variables, therefore radiomic features, compared to the number of samples, leading to difficulty for ML models to learn effectively (49). Hence, suggesting the potential for multimodal data integration to overcome practical ML model implementation challenges in clinical settings.

### 4.5 Strengths and limitations

The review benefitted from having two independent reviewers involved in the study selection, data extraction, and result interpretation processes. This dual-independent screening approach helps minimise potential bias and enhance the reliability and validity of the findings. Having two reviewers also allowed for cross-checking and helped mitigate individual biases.

The quality and RoB assessment using QUADAS-2 revealed low concerns regarding applicability but suggested unclear RoB, which led to some concerns overall. Methodological details regarding the index test, including blinding and predetermining threshold during the use of ML models, were often unclear. The patient selection methods detailed often suggested a consecutive selection process by searching through databases and specifying the timeframe in which patients were tested. Nonetheless, the studies lacked explicit documentation of consecutive or random recruitment. The unclear reporting of the methodology is a limitation consistently observed in the literature evaluated in this review therefore impacting the RoB for Domains 1 and 2, affecting overall study quality. These findings are consistent with the results of the conducted METRICS appraisal (Table 6). The METRICS score ranged from 67.7% to 89.7%, with most of the appraised studies being evaluated as being “Good” quality with only two studies being evaluated as “Excellent” quality (30, 37). Higher-scoring radiomic studies provided clearer methodological descriptions and more robust validation methods, such as the inclusion of external validation sets. Overall, the findings from the QUADAS-2 and METRICS appraisal emphasise the importance of methodological transparency to improve the reliability of radiomics and ML studies in predicting glioma mutation status (23).

A limitation of this review was that only two studies used external validation (30, 37). External validation strengthens evidence regarding model robustness while also demonstrating greater generalisability and applicability in a variety of clinical settings. Most of the included studies employed internal cross-validation techniques to mitigate overfitting, where ML models become overly familiar with the training data, including its outliers. As a result, the MLA performs exceptionally on training data but performs poorly on unseen datasets due to over-reliance on memorised data rather than identifying underlying patterns to predict the presence of IDH mutations and ATRX loss. Cross-validation divides datasets into subsets to train models on different combinations and expose MLA to new test data. This ensures consistent performance across the various subsets of the internal data. Nonetheless, cross-validation cannot fully assess the predictive capabilities of models on completely independent datasets. Future research should aim to include larger datasets and allow for external validation to better evaluate the generalisability and clinical utility of predictive models for clinical implementation.

Integrating ML into clinical practice for the management of glioma involves significant initial costs, such as technical training for staff, acquiring required technological infrastructure, and advanced imaging tools. However, in the long term, these investments may reduce the need for invasive procedures and frequent diagnostic testing, thereby improving clinical workflow and reducing overall costs. By balancing short-term expenses with potential long-term savings and improved patient outcomes, this review highlights the strength of this approach in glioma management (50).

### 4.6 Clinical implications and future recommendations

This systematic review highlights the potential of ML models to provide a powerful, non-invasive approach for predicting IDH and ATRX mutations using radiomics from conventional MRI. This approach enhances diagnostic precision, facilitating early identification of glioma biomarkers and improving personalised treatment outcomes. High diagnostic accuracy for IDH mutations supports integrating ML models into clinical practice to aid clinicians in diagnosis and decision-making. However, the lower sensitivity for detecting ATRX loss indicates the need for further research to improve glioma classification and clinical application. These clinical implications align with Singh et al. (38), who emphasised that advances in radiomics offer less invasive approaches to glioma diagnosis, thereby, informing surgical planning and therapeutic strategies. Therefore, these advances in radiomics refine glioma management and optimise patient care.

Significant potential benefits can be offered through the integration of ML with the predicting of IDH and ATRX mutations into clinical practice. Potential benefits include reduced reliance on invasive biopsies for diagnosis, enhanced diagnostic accuracy, and early detection of mutations in glioma patients, ultimately, leading to timely and targeted treatments. However, careful consideration of various factors is imperative in the implementation of this approach. This involves considering the economic viability of implementing ML and addressing technical challenges. These challenges include standardising data collection and processing procedures across various organisations while ensuring data privacy and security when handling large amounts of sensitive patient information. Resistance to change may be another barrier to the implementation of this approach. Therefore, it is crucial to gain clinical acceptance by gaining trust from clinicians who may be hesitant to rely on the incorporation of ML models with neuroimaging over traditional molecular diagnostic methods. Additionally, considerations around regulatory and ethical concerns, including navigating the approval processes for implementing ML-based tools, addressing algorithm bias and ensuring equitable access to advanced diagnostic technologies, should be addressed (51, 52). Future research should focus on standardising data collection, clarifying methodologies and improving ML model validation to increase reliability across diverse clinical settings. The review also highlights that incorporating patient demographics and semantic features with radiomic data can further enhance the predictive capabilities of ML models. This holistic approach combines imaging and clinical data to create robust diagnostic tools tailored for clinical application. Integrated models, which include clinical characteristics and combine conventional MRI with advanced imaging modalities, show promise. Future research should address the standardisation of multimodal data collection and validate these enhanced models by conducting multicentred cohorts to ensure generalisability (47).

Furthermore, the use of METRICS, which is a recently introduced appraisal tool, has been highly efficient and relevant in the evaluation of the methodological quality of studies of this nature (23). Therefore, it significantly supports the reliability of the appraisal process in this systematic review. Incorporating the METRICS tool in future research is recommended to ensure robust evaluations and enhance reliability when quality appraising methodological rigor and transparency in radiomics and ML research.

In addition, although integrating ML into the diagnostic and treatment planning processes for glioma patients requires a substantial initial investment, including acquiring advanced imaging tools and establishing ML infrastructure, this approach holds significant potential for long-term cost reductions by reducing the need for invasive procedures and enabling more precise treatment planning (50–52). Therefore, it will be crucial to perform cost–benefit analyses to facilitate the widespread adoption of advanced ML models into clinical practice. Barriers to the integration of ML into molecular identification and prediction for glioma patients include the necessity for specialised training for clinical practitioners, integrating new workflows and procedures into existing clinical operations and ensuring privacy and security are upheld when handling sensitive patient information (50). Overcoming these barriers requires effective cooperation among healthcare professionals, professional bodies, health legislators and technology developers to establish standardised protocols. Additionally, this collaboration should ensure the provision of necessary resources and training to support the integration of ML-based approaches in predicting molecular status and diagnosing glioma patients. Identifying and predicting molecular status in glioma patients efficiently and accurately can improve glioma classification. Therefore, overcoming barriers to the widespread integration of ML in the diagnostic pathway for glioma patients can lead to improved diagnostic accuracies, making targeted therapies more feasible and overall, improved patient outcomes.

## 5 Conclusion

This systematic review highlights the high and moderate accuracy of ML models in predicting IDH and ATRX mutation statuses in gliomas, respectively. Recent studies correlate ATRX loss and IDH mutations with improved prognosis due to enhanced immune response and increased genomic instability. Both are key diagnostic genes in the 2021 WHO Classification of CNS Tumours. The current gold standard for glioma classifications is histopathological diagnosis via invasive procedures including biopsy or tumour resection, which carry inherent risks such as infection and bleeding. These recent developments therefore strengthen the significance of this review. Combining neuroimaging with ML approaches shows promise in the accurate classification and prediction of glioma mutations. This approach demonstrates the potential to reduce the need for invasive diagnostic procedures and improve patient outcomes through lower-risk yet early and precise diagnosis (1, 7).

The meta-analysis showed that ML had higher pooled sensitivity and AUC for predicting IDH mutations compared to ATRX loss, indicating proficiency in identifying IDH mutations. However, further improvements in ML performance in predicting ATRX loss are suggested. Additionally, incorporating patient demographics and semantic features into ML models significantly improves accuracy and clinical relevance. Findings and recommendations for future research made in this review will contribute to clinical adoption, enhancing patient outcomes through precise, non-invasive and individualised diagnosis, prognosis and treatment plans.

## Data Availability

The datasets presented in this study can be found in online repositories. The names of the repository/repositories and accession number(s) can be found in the article/Supplementary Material.
