# Supplementary material for: Predicting IDH and ATRX mutations in gliomas from radiomic features with machine learning: a systematic review and meta-analysis
Source: Front Radiol. 2024 Oct 31;4:1493824. doi: 10.3389/fradi.2024.1493824 (PMC11560782; doi:10.3389/fradi.2024.1493824)
Supplement: Supplementary file 1 [file Datasheet1.docx]

**Supplementary Material**

| Abbreviations | Full terminology | Explanation |
| --- | --- | --- |
| ATRX | Alpha-Thalassemia/Mental Retardation Syndrome X-Linked | A gene is involved in essential cellular pathways such as chromatin remodeling. ATRX loss are key biomarkers in glioma diagnosis and can influence prognosis and tumor behaviour. |
| ALT | Alternative Lengthening of Telomeres | A telomere maintenance mechanism that can be activated in cancers cells, which contributes to cancer cell immortality by avoiding telomere degradation. |
| ASL | Arterial Spin Labeling | A magnetic resonance imaging method that measures cerebral blood flow for patients with glioma. |
| AUC | Area Under the ROC Curve | An accuracy metric for quantitative diagnostic tests and predictive models. Higher values indicating better performance as opposed to lower values indicating poorer performance. |
| BR | Binary Relevance | A multi-label classification technique that treats each label as an independent binary classification problem. Classifying each label as relevant or irrelevant. |
| C3D | Convolutional 3D | A deep learning model in which convolutional operations are applied in three dimensions. Often used to analyze volumetric data. |
| CBF | Cerebral Blood Flow | A measure of the rate of perfusion to brain tissue. Indicating the blood volume that flows to a given amount of brain tissue per unit of time. |
| CNN | Convolutional Neural Network | A type of deep learning algorithm particularly effective for analyzing visual data such as image recognition tasks and processing. |
| DWI | Diffusion-Weighted Imaging | An MRI sequence that detects proton movements in water molecules allowing the measuring of the diffusion of water molecules in tissue. |
| EC | Ensemble Classifier | A model in which predictions from multiple algorithms are combined to produce improved results. |
| FLAIR | Fluid-Attenuated Inversion Recovery | A special inversion recovery sequence that detects lesions and nullifies cerebral fluid signals in the brain. |
| HARDI | High Angular Resolution Diffusion Imaging | An advanced MRI technique that can provide detailed images of brain’s white matter structures by accurately mapping the complex neural pathways within the white matter tracts. |
| IDH | Isocitrate Dehydrogenase | An enzyme that plays key roles in cellular metabolism. IDH mutations occur in some gliomas and are associated with clinical prognosis. |
| ML | Machine Learning | A subtype of artificial intelligence focusing on the use of data and algorithms to automatic learn from past experiences such as in identifying patterns and making predictions and gradually improve accuracy. |
| MRI | Magnetic Resonance Imaging | A type of scan and medical imaging technique where magnetic fields and radio waves are used to visualize detailed internal structures. |
| PEO | Population, Exposure, Outcome | A framework to support the formulating of research questions and guide the planning of search strategies. |
| PICOT | Population, Intervention, Comparison, Outcome, Time | A framework used to support the defining of inclusion and exclusion criteria in systematic reviews. |
| QSM | Quantitative Susceptibility Mapping | An MRI technique that quantifies the magnetizations tissues induce, reflecting tissue magnetic susceptibility. |
| ResNet50 | 3D residual deep neural network 50 | A deep learning model with a 50-layer convolutional neural network. The model is used for image classification and can be trained on large datasets. |
| RF | Random Forest | A machine learning algorithm that is commonly used to reach a single result by combining outputs from multiple built decisions trees. |
| SVM | Support Vector Machine | A supervised learning algorithm used for the classifying of data by finding the optimal hyperplane, which is the decision boundary that best separates different classes. |
| SWI | Susceptibility-Weighted Imaging | An MRI sequence is used to visualize blood products, calcium or other compounds that distort the magnetic field. |
| T1 | T1-weighted MRI | A basic pulse sequence in MRI that provides high-resolution images of anatomical detail. |
| T2 | T2-weighted MRI | A basic pulse sequence in MRI enhancing signal of water, therefore effective in detecting fluid and pathology. |

**Figure S1.** Abbreviations and explanations table.

**Data extraction form**

**Title of systematic review:** Use of machine learning in the prediction of ATRX and IDH mutation status in glioma: A Systematic Review and Meta-Analysis

**1. General information**

| **Author** | Evan Calabrese, Javier E. Villanueva-Meyer, Soonmee Cha |
| --- | --- |
| **Year of publication** | 2020 |
| **Title of study** | A fully automated artificial intelligence method for non-invasive imaging-based identification of genetic alterations in glioblastomas. |

**2. Study Characteristics**

| **Study design** | Retrospective analysis. |
| --- | --- |
| **Setting** | Single centre study at University of California, San Francisco. |
| **Duration of study** | 2015 to 2019. |
| **Study funding source(s)** | National Institutes of Health Ruth L. Kirschstein Institutional National Research Service Award and University of California San Francisco resident research fund. |

**3. Population**

| **Sample size** | 199 patients. |
| --- | --- |
| **Population and target condition** | Adults with histopathologically confirmed grade IV malignant glioma (glioblastoma). |
| **Inclusion criteria** | Histopathologically confirmed glioblastoma by genetic sequencing or immunohistochemical staining at the time of biopsy or resection, having had preoperative MRI. |
| **Exclusion criteria** | History of prior brain tumour diagnosis or treatment. |

**4. Machine Learning Model (Index Test)**

| **Index test description** | Extracted radiomic features from MRI scans analysed using ML approach to for presence of each genetic biomarker. |
| --- | --- |
| **Type of ML** | Random forest regression models. |
| **Data source** | Preoperative brain MRI of patients with glioblastoma. |

**5. Reference Standard**

| **Reference standard description** | Genetic sequencing and/or immunohistochemical staining carried out at the time of biopsy or tumour resection. |
| --- | --- |
| **Tested Biomarkers** | IDH, ATRX, TP53, PTEN, TERT, CDKN2, MGMT promoter methylation, EGFR, 7/10 aneuploidy. |

**6. Types of Outcome Measures**

| **Primary outcome** | Accuracy of predicting genetic biomarkers from radiomic features using deep learning segmentation in glioblastoma patients. |
| --- | --- |
| **Secondary outcome** (If applicable) | Performance characteristics including sensitivity and specificity of the ML algorithm. |

**7. Results**

| **Accuracy metrics compared to reference standard** | **Overall accuracy** | **AUC** | **Sensitivity** | **Specificity** |
| --- | --- | --- | --- | --- |
| **ML accuracy in predicting IDH mutations** (AUC, sensitivity and specificity) | High | 0.95 ± 0.03 | 0.93 ± 0.08 | 0.88 ± 0.07 |
| **ML accuracy in predicting ATRX mutations** (AUC, sensitivity and specificity) | High | 0.97 ± 0.02 | 0.94 ± 0.07 | 0.92 ± 0.04 |

**8. Discussion and Conclusion**

| **Key conclusions drawn by author** | The machine learning approach has been shown to be effective in the prediction of genetic biomarkers in patients with glioblastoma patients as a non-invasive method. |
| --- | --- |
| **Limitations** | Limited generalizability. Reliance on tools not involved in routine clinical practice including 3 T MR scanners, 3 D imaging and 55-direction HARDI, certain biomarkers positive only in a small subset of the cohort. Single centre study limiting generalizability to other patient demographic groups. |
| **Strength** | Imbalances in dataset addressed with a stratified cross-validation approach. Use of automated extraction technique improves reproducibility of the study. Retrospective analysis allowing comprehensive analysis of existing data. |
| **Remarks** | Potential in detection of glioblastoma biomarkers non-invasively using radiomics and machine learning for an automated approach to guide therapy and determine prognosis. |

**Figure S2.** An example of a completed data extraction form. Created using Microsoft Word to add specific questions to address the aims and objectives of the systematic review. Completed regarding Calabrese, Villanueva-Meyer & Cha, 2020’s study.


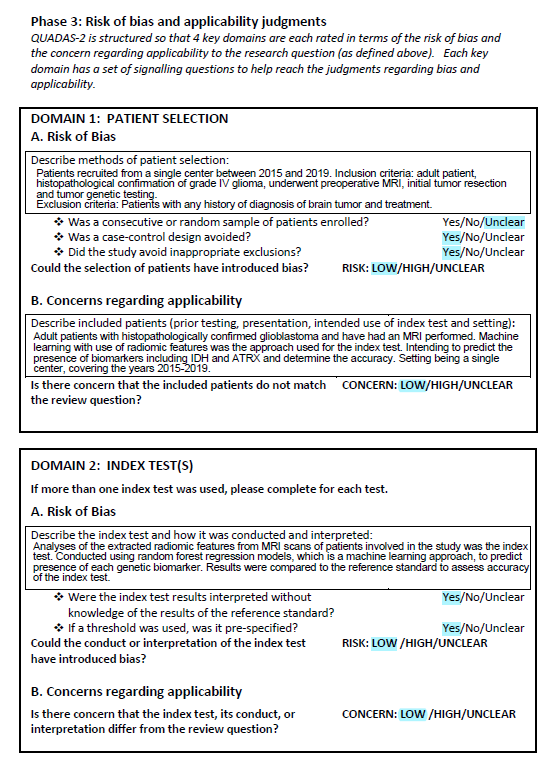

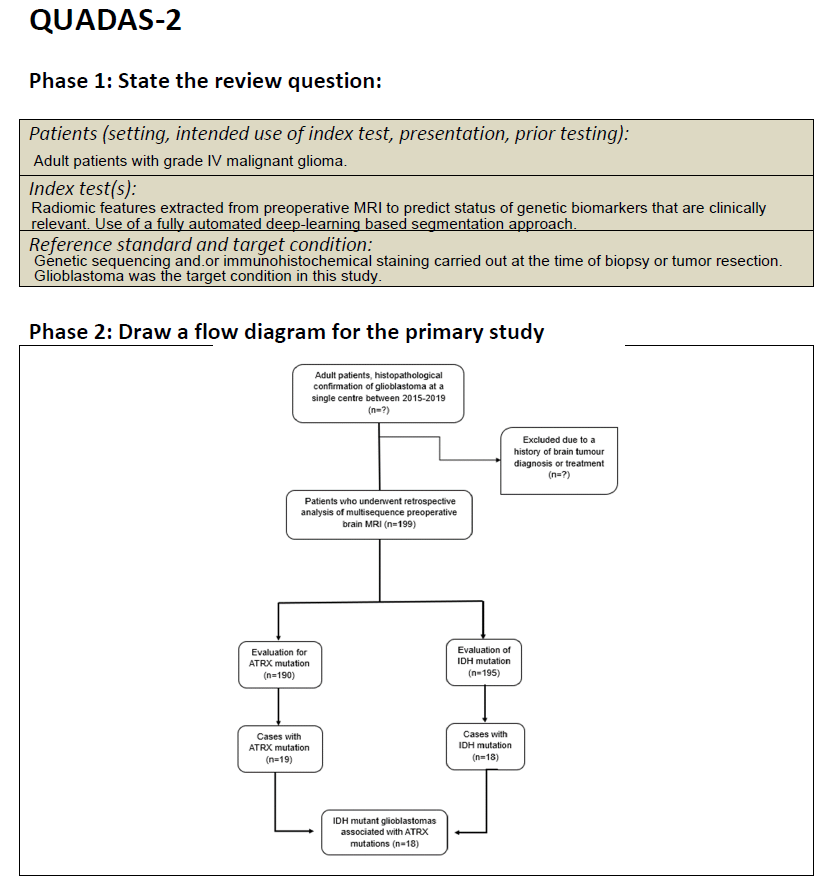


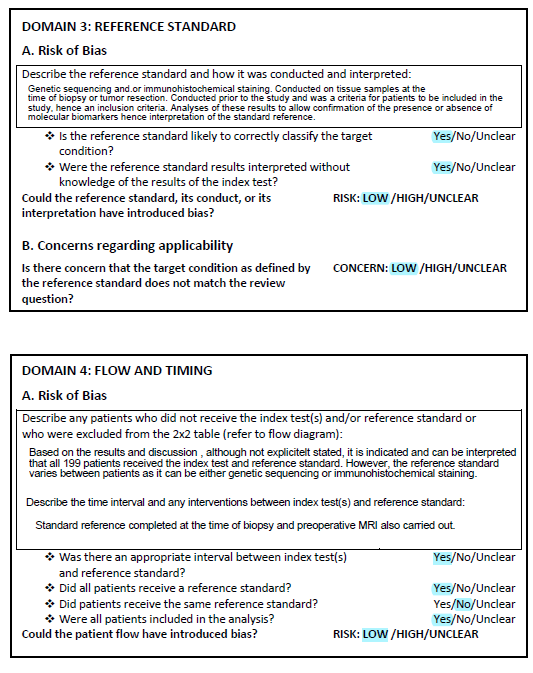


**Figure S3.** Example of a completed quality appraisal form using the QUADAS appraisal tool. Completed regarding Calabrese et al., 2020’s study.

**Table S1.** Reconstructed 2 x 2 confusion matrix for the performance of ML models in predicting IDH mutations.

| **Study ID** | **Sample size** | **Number of IDH mutations** | **TP** | **FN** | **FP** | **TN** |
| --- | --- | --- | --- | --- | --- | --- |
| Ren 2019 | 57 | 36 | 34 | 2 | 3 | 18 |
| Calabrese 2020 | 195 | 18 | 17 | 1 | 21 | 156 |
| Shboul 2020 | 108 | 85 | 77 | 9 | 5 | 18 |
| Sohn 2021 (BR) | 126 | 5 | 5 | 0 | 14 | 107 |
| Sohn 2021 (EC) | 126 | 5 | 5 | 0 | 14 | 107 |
| Haubold 2021 | 164 | 81 | 56 | 25 | 17 | 66 |
| Calabrese 2022 | 400 | 29 | 25 | 4 | 22 | 349 |
| Rui 2023 | 41 | 23 | 20 | 3 | 6 | 12 |
| Zhong 2023 (3DResNet) | 33 | 3 | 2 | 1 | 8 | 22 |
| Zhong 2023 (C3D) | 33 | 3 | 3 | 0 | 5 | 25 |

**Table S2.** Reconstructed 2 x 2 confusion matrix for the performance of ML models in predicting ATRX mutations

| **Study ID** | **ATRX size** | **N.O. ATRX mutations** | **TP** | **FN** | **FP** | **TN** |
| --- | --- | --- | --- | --- | --- | --- |
| Ren 2019 | 36 | 19 | 18 | 1 | 2 | 15 |
| Calabrese 2020 | 190 | 19 | 18 | 1 | 13 | 158 |
| Shboul 2020 | 108 | 43 | 30 | 13 | 11 | 54 |
| Sohn 2021 (BR) | 126 | 17 | 9 | 8 | 5 | 104 |
| Sohn 2021 (ECC) | 126 | 17 | 12 | 5 | 16 | 93 |
| Haubold 2021 | 67 | 13 | 9 | 4 | 8 | 46 |
| Calabrese 2022 | 396 | 34 | 33 | 1 | 43 | 319 |
| Rui 2023 | 22 | 11 | 7 | 4 | 5 | 6 |
| Zhong 2023 (3DResNet) | 36 | 3 | 2 | 1 | 5 | 28 |
| Zhong 2023 (C3D) | 36 | 3 | 3 | 0 | 3 | 30 |
